# Assessing the effectiveness of group motivational interviewing in raising awareness of mobile gaming addiction among medical students: a pilot study

**DOI:** 10.1186/s13104-025-07250-y

**Published:** 2025-04-16

**Authors:** Leonard Yik Chuan Lei, Yoke Yong Chen, Chee Shee Chai, Keng Sheng Chew

**Affiliations:** Department of Medical Education, Faculty of Medicine and Health Sciences, Universiti Malaysia Sarawak (UNIMAS), Sarawak, 94300 Kota Samarahan Malaysia

**Keywords:** Mobile game addiction, Group motivational interviewing, Medical students

## Abstract

**Objective:**

Group Motivational Interviewing may raise awareness of mobile gaming addiction. MI has reported reduction of gaming addiction in adolescents, although its effectiveness among medical students remains underexplored. This study assessed the effectiveness of group MI in raising awareness of mobile gaming addiction among medical students.

**Results:**

Significant progression in Stages of Change at pre- to post-intervention (χ² = 41.891, *p* < 0.001; Cramer’s V = 0.555) and from post- to two-months post-intervention (χ² = 87.083, *p*-value < 0.001; Carmer’s V = 0.800). IAIM scores improved over time (χ² = 9.349, *p* = 0.009), with the highest improvement at two-months. A moderate positive correlation (ρ = 0.517, *n* = 34, *p* < 0.002) was found between self-reported and mobile game usage at two-months. This pilot study provides early evidence that GMI may enhance motivation to reduce mobile gaming and support progression through stages of change. Future studies could employ larger randomized controlled trials (RCT) with longer follow-up periods.

**Trial registration:**

International Standard Randomised Controlled Trial Number (ISRCTN) Registry ISRCTN93544148. Date of registration 05/02/2025. Retrospectively registered.

**Supplementary Information:**

The online version contains supplementary material available at 10.1186/s13104-025-07250-y.

## Introduction

Gaming has shifted to mobile games from desktop and console games [1]. Lee and Kim [2] describe mobile games as portable, instant and readily available. In contrast, desktop and console games may contribute to addiction via complex gameplay and time-based rewards [3]. A recent meta-analysis reported the prevalence of internet gaming disorder (IGD) at 6.2% [4] among medical students, nearly twice of the general population (3.05%) [5]. Previous studies show depression and poor academic performance are linked to mobile game addiction [6, 7] and physical symptoms blurred vision and headaches [8].

Group motivational interviewing (GMI) delivers MI in groups to increase motivation and promote behavioural change via collaboration [9]. Previous MI studies on gaming addiction have focused primarily on adolescent groups [10, 11]. Medical students were selected due to higher academic pressures, longer study periods [12], and higher rates of addictive behaviours [13]. This study assessed the effectiveness of group MI in raising awareness of mobile gaming addiction among medical students.

## Method

**Fig. 1:**
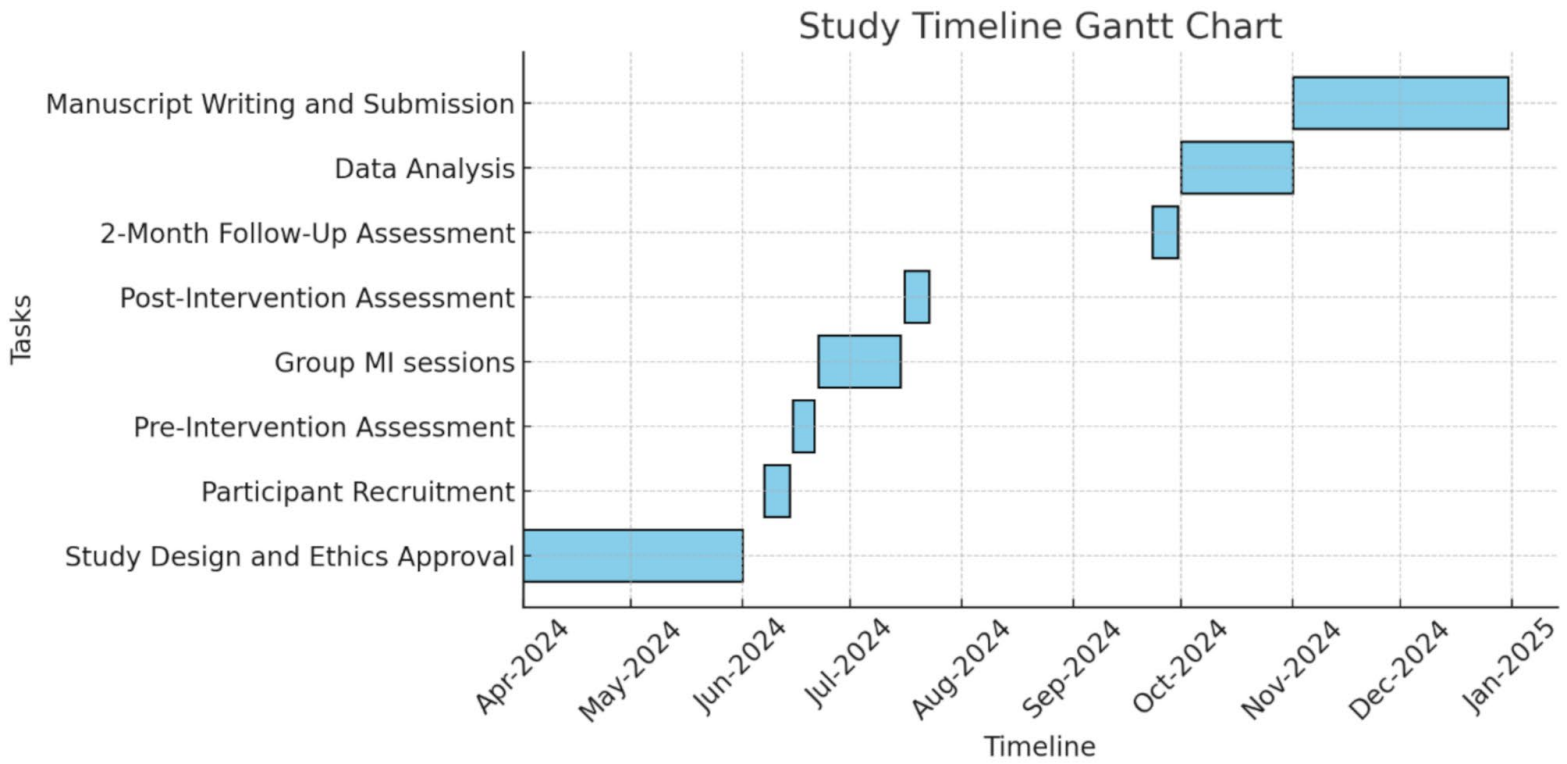
This Gantt chart illustrates the study timeline which includes the study design, ethics approval, recruitment, intervention, data collection and analysis, and manuscript writing between April 2024 and December 2024

A single arm pilot study, pre-post with two-months follow-up was conducted to assess GMI’s effectiveness in raising awareness of mobile gaming addiction among medical students. Given the explorative nature of this study, a single arm approach was selected to examine the potential effects of GMI prior to performing large scale-controlled trials. A study timeline Gantt chart is presented in Fig. 1.

### Participants

Medical students with smartphones or tablets were included. Exclusion criteria were learning disabilities, the history of substance abuse or psychotherapy for IGD for the past 6 months, suicidal ideation. Participants with prior MI training and psychotherapy were excluded to minimize confounding factors due to varying baseline levels of readiness to change.

### Procedure

Third year medical students recruited from the Faculty of Medicine and Health Sciences (FMHS), at Universiti Malaysia Sarawak (UNIMAS) were recruited via in- person class announcements and official WhatsApp groups. After providing written consent, three sessions of GMI were conducted over the course of three weeks. Evaluations were conducted at T0 (pre-intervention) (0 weeks) (T0), post-intervention (3 weeks) (T1), and follow-up two-months post-intervention (T2).

### Intervention

The intervention was GMI implemented during a medicine posting rotation for third-year medical students. A total of three sessions were conducted, each lasting one hour, held over a period of three weeks. Intervention content was adapted from Wagner and Ingersoll [14] model of GMI and delivered by first author, who completed 60-hour MI for addiction workshop and was supervised by CYY. The content was adapted to third year medical students by addressing academic stress, gaming habits, peer influence, and time management challenges. The content was reviewed by KSC and by CYY, a trained clinical psychologist with expertise in MI to ensure intervention integrity. All participants received GMI handouts. No control group was included. The details of the program are shown in Table 1.

**Table 1:** Session’s descriptions

Session	Content	Goal	MI techniques	Duration
Session 1 (First Week)	Phase 1 (Engaging the Group) Introduction Main Objectives, Approaches, Expectations Group Guidelines Eliciting Goals	To foster positive and collaborative setting to enhance dialogue about positive behavioural change	Open Ended Questions, Affirmations, Reflective listening, Summary (OARS) were used to promote engagement and establish group communication.	1 h
	Phase 2 (Exploring Perspectives) Lifestyles and Habits Ambivalence Exploring Values	To help participants in reframing their stressors within a broader context of their lives through the process of examining their habits, core values, lifestyle, ambivalence	OARS, evoking change talk were utilized to support participant’s reflection on their motivations, and their ambivalence.	
Session 2 (Second Week)	Phase 3 (Broadening Perspectives) Develop Discrepancy Decisional Balances Looking towards the future Strengths & Success Stories	To assist in broadening participant’s perspectives to guide them in envisioning a brighter future	OARS, decisional balance exercises, developing discrepancy were applied to assist in identifying the contrast between their current behaviours and desired outcomes	1 h
Session 3 (Third Week)	Phase 4 (Moving into Action) Importance and Confidence Review Change Planning Strengthening Commitment to Change Dealing with Challenges and Setbacks	To facilitate in defining, working on and carrying out changes that participants view could improve their lives	OARS, goal setting, importance and confidence rulers were used to empower participants and enhance self-efficacy to make positive changes.	1 h

This intervention of GMI was adapted based on Wagner and Ingersoll [14] model for carrying out MI in groups.

### Primary outcomes

#### Stages of change progression

The stages of change progression was assessed through the Adapted Stages of Change (SoC) questionnaire [16] (see Supplementary File 1). This study will measure stages of change via a single item: “Did you play mobile games for 20 hours or more a week during last month?“. Single item measures have been used in behavioral research articles [16, 17] for their feasibility and practicality in evaluating stages of change transitions. While multi-item instruments provide enhanced psychometric rigor, single item instruments are effective in assessing observable behavioral constructs like stages of change.

#### Motivation to improve mobile game addiction

Motivation to improve mobile game addiction was assessed via Internet Addiction Improvement Motivation Scale (IAIMS) [18], a 10 item, 6-point Likert scale. High risk groups with low motivation were identified by scores below 33 or by subscale scores less than 10, 11 and 9 for precontemplation, contemplation, and preparation respectively. The internal consistency for these subscales was reported to be 0.613, 0.724 and 0.734 confirming acceptable reliability.

### Secondary outcomes

#### Relationship between self-reported and mobile game usage

Self-reported mobile game addiction was measured via Internet Gaming Disorders Scale– Short Form (IGDS9-SF) [19], 9 items, with a 5-point Likert scale from 1 (Never) to 5 (Very often). Disordered gamers minimum was indicated with a score of 36/45 points. Internal consistency was acceptable (α = 0.87).

#### Mobile game usage

Screen Time [20] and Digital Wellbeing [21] were used to evaluate mobile game usage on smartphones and tablets. These apps did not record, store data or modify the applications. Participants manually recorded their mobile game usage in total weekly mobile game usage (in minutes) at each time point.

### Sample size

Sample size was calculated based on a prior study [22] of group cognitive behavioural therapy (CBT) for Internet Addiction, demonstrating large effect size (Cohen’s d = 1.08). Utilizing G*Power software [23], with a power of 0.95, significant level of 0.05, the calculated sample size was 24 per group. This was adjusted to 34 per group to account for 30%, dropout rate. Convenience sampling was used. Post hoc power analysis was 83.4% power (df = 2) for pre-vs post-intervention and 99.1% (df = 2) for post-intervention vs. two-months follow-up.

#### Analysis

Data were analysed via Statistical Package for Social Sciences (SPSS) version 29. Normality of IAIMS was assessed via Shapiro-Wilk test. The assumption of normality was met at pre-intervention and two-months post-intervention (*p* = 0.353 and *p* = 0.097), but not at post intervention (*p* = 0.018). Chi-square tests assessed the relationship between stages of change across time points (pre-post, post-follow-up). The Friedman test and Kendall’s coefficient of concordance (Kendall’s W) [15] measured the effects of GMI on IAIMS scores over three time points. Spearman correlations examined the relationship between self-reported mobile game addiction and objectively measured mobile game usage.

## Results

**Fig. 2:**
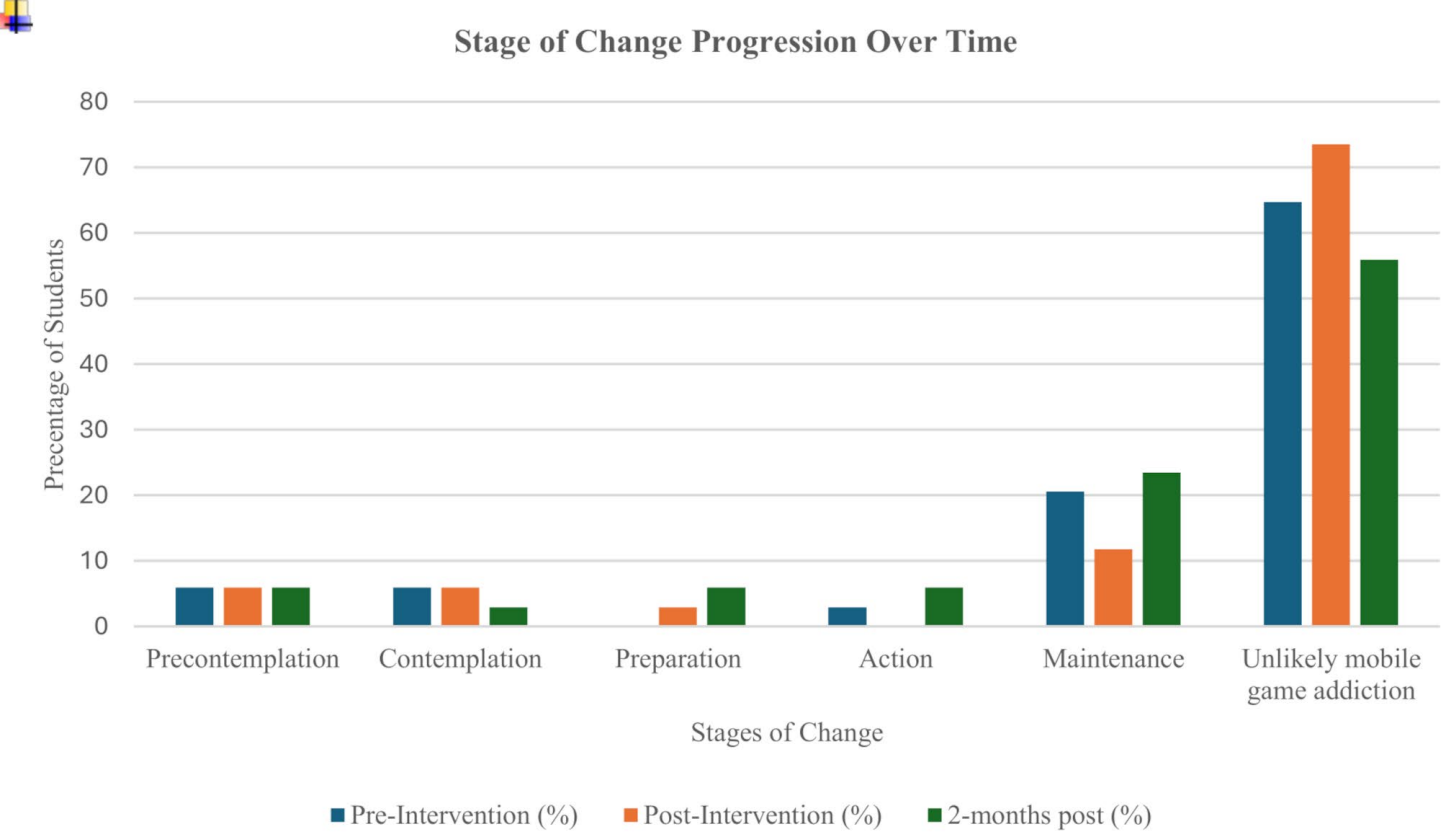
Shows the distribution of students across the stages of change at pre-intervention, post-intervention and two-months post-intervention. A noticeable shift of students from the contemplation stage towards maintenance and unlikely mobile game addiction, suggests the impact of GMI on the readiness to change

Out of the 40 students approached, 38 (95%) students completed all three GMI sessions, but only 34 (85%) students completed all sessions and questionnaires. Those who missed the questionnaires (*n* = 4; 10%) or sessions were excluded (*n* = 2; 5%), resulting in 34(85%) students included in the final analysis. Reasons for non-completion were due to personal scheduling conflicts and illness. The mean age was 22.15 years (SD = 0.36). Most participants were females (*n* = 24; 70.6%), followed by males (*n* = 10; 29.4%). Ethnic distribution: Malay (*n* = 15; 44.1%), Bumiputera Sarawak (*n* = 9; 26.5%), Chinese (*n* = 8; 23.5%), and Indian (*n* = 2; 5.9%). Figure 2 shows the distribution of students across the stages of change at pre-, post-and two-month follow-up.

### Primary outcome

#### Pre-intervention vs. post-intervention

The Pearson Chi Square test demonstrated a significant shift between pre-intervention and post-intervention stages of change (χ² = 41.891, *p* < 0.001). Cramer’s V = 0.555 (*p*-value < 0.001) revealed a moderate to strong association (Table 2). This suggested that the student’s readiness to change shifted significantly post intervention.

**Table:** Table 2

Cramer’s V Value	Effect Size Interpretation
0.07–0.21	Small Effect
0.21–0.35	Medium Effect
> 0.35	Large Effect

Adapted from Cohen [44], effect size interpretation for chi-square tests with df = 2

#### Post-intervention vs. two-months post-intervention

The Pearson Chi Square test showed a significant relationship between post and two-months post-intervention stages of change (χ² = 87.083, *p*-value < 0.001), suggested continued participant’s progression. Cramer’s V = 0.800 (*p* < 0.001) showed strong association. This suggested that the intervention sustained and improved readiness for change.

#### IAIMS scores

IAIMS mean ranks (1.60, 2.09, 2.31) indicated a statistically significant difference over time (χ² = 9.349, *p* = 0.009), and a small effect size (Kendall’s W = 0.1375). GMI significantly enhanced participants motivation with the highest improvement at two-months post-intervention.

### Secondary Outcome

#### Pre-intervention correlation between IGDS9-SF and game usage

A moderate positive correlation was pre-intervention between IGDS9-SF and game usage (ρ = 0.601, *n* = 34, *p* < 0.001, Table 3), suggesting that higher pre-intervention mobile game addiction was associated with higher mobile game usage.

**Table 3:** Spearman correlation between reported mobile game addiction level and game usage

Time Point	ρ (Spearman Coefficient)	*P*-value	Strength of Correlation	*N*	Interpretation
Pre-intervention	0.601	< 0.001	Moderate positive	34	Moderate positive correlation, statistically significant
Post-intervention	0.399	0.019	Moderate positive	34	Weaker than pre-intervention, statistically significant
Two-months post-intervention	0.517	0.002	Moderate positive	34	Slightly stronger post intervention, but weaker than pre-intervention, statistically significant

#### Post-intervention correlation

A moderate positive correlation was observed post-intervention between IGDS9-SF and game usage (ρ = 0.399, *n* = 34, *p* < 0.019), suggesting that higher post-intervention mobile game addiction levels were associated with higher mobile game usage. However, this relationship was weaker compared to pre-intervention.

#### Two-months post-intervention correlation

A moderate positive correlation was found two-months post-intervention between IGDS9-SF and game usage (ρ = 0.517, *n* = 34, *p* < 0.002), suggesting that higher mobile game addiction levels were associated with higher mobile game usage. However, this association was weaker compared to pre-intervention.

## Discussion

To the best of our knowledge, this is the first study to assess the effectiveness of GMI in raising awareness of mobile gaming behaviours among medical students. A significant relationship was observed between stages of change pre- to post and two-months follow-up. Our results were consistent with previous studies suggesting MI improves readiness to change in behavioural addictions [24, 25, 26, 27]. One possible explanation is that MI fosters self-efficacy (defined as the confidence in an individual’s ability to modify health behaviours [28]), which facilitates progression through the stages of change.

Several studies [11, 29, 30, 31, 32, 33] have utilized MI to reduce internet gaming behaviours. Tse, Siu [31] conducted GMI via a mixed methods approach across primary, secondary and university students and found reductions in gaming time and enhanced motivation, though risks of IGD remained unchanged. These findings align with our study, which showed improved motivation to improve mobile game addiction. In an RCT, Nuryono [30] reported that Family Counselling MI approach more effective than cognitive behavioural therapy (CBT) in reducing game addiction among adolescents. Similarly, Kaur and Dhillon [32] observed that MI interventions improved attitude and behavioural outcomes for IGD in adolescents. Deep learning models [34, 35, 36, 37, 38, 39] may help predict and diagnose the risks of mobile game addiction, informing MI strategies.

IAIMS scores indicated significant difference over time with small effect size, suggesting that GMI improved students’ motivation to improve mobile game addiction, with the highest improvement at two-months post-intervention. This could be due to GMI’s group approach, which fosters a supportive environment for shared learning [40].

The moderate positive correlation between self-reported and application recorded mobile game usage at two-months post-intervention suggests changes were not sustained in the long-term. The moderate correlation also suggested that both measures are related but not fully aligned, potentially affecting measurement accuracy. Our findings align with previous studies reporting positive correlation between problematic gaming and time spent gaming [41, 42]. According to self-determination theory [43] fulfilling the needs for autonomy, competence and relatedness may account for this outcome.

### Limitations

Limitations include the use of a single cohort of third year medical students and purposive sampling, which may affect the generalizability across academic years and disciplines. Students from other disciplines or age groups may respond differently. The small sample size may reduce the reliability of statistical analysis. The absence of a control group in this study limits causal interpretation and increases susceptibility to confounding factors such as academic and exam schedules, seasonality and social influences. Reliance on self-reports introduces a risk of social desirability and recall bias. Response variability may be due to differences in motivation, engagement and personal gaming behaviour. Future studies could incorporate third-party assessments to strengthen the objectivity of the results.

## Conclusion

This pilot study provides early evidence that GMI may enhance motivation to reduce mobile gaming and support progression through stages of change. Integration into student wellness programs may be considered. Future studies could employ larger randomized controlled trials (RCT) with longer follow-up periods.

## Electronic supplementary material

Below is the link to the electronic supplementary material.

Supplementary Material 1

## Data Availability

Data generated and analysed during the current study are not publicly available as individual privacy could be comprised; however, may be available from the corresponding author on reasonable request and with the permission of the medical school.

## Abbreviations

SOC: Adapted stages of change
CBT: Cognitive Behavioural Therapy
FMHS: Faculty of Medicine and Health Sciences
GMI: Group motivational interviewing
IAIMS: Internet addiction improvement motivation scale
IGD: Internet gaming disorder
IGDS9-SF: Internet gaming disorders scale– short form
Kendall’s W: Kendall’s coefficient of concordance
MI: Motivational interviewing
OARS: O = open ended questions, A = Affirmations, R = Reflections, and S = summaries
RCT: Randomized controlled trials
SPSS: Statistical package for social sciences
UNIMAS: Universiti Malaysia Sarawak
