# Supplementary material for: Assessing the effectiveness of group motivational interviewing in raising awareness of mobile gaming addiction among medical students: a pilot study
Source: BMC Res Notes. 2025 Apr 16;18:178. doi: 10.1186/s13104-025-07250-y (PMC12004664; doi:10.1186/s13104-025-07250-y)
Supplement: Supplementary file 1 — Supplementary Material 1 [file 13104_2025_7250_MOESM1_ESM.docx]

**Research topic: Assessing the effectiveness of group motivational interviewing in raising awareness of mobile gaming addiction among medical students: A pilot study**

**Please fill in the required information below:**

| ID number |  |
| --- | --- |
| Year of Study |  |
| Age |  |
| Gender |  |
| Ethnic Group |  |

**Bergen Social Media Addiction Scale**

The following questions will ask about your general social media activities. Please read each statement and circle a number 1 to 5 which indicates how much the statement applied to you over the past year. There are no right or wrong answers. Do not spend too much time on any statement.

|  | Very Rarely | Rarely | Sometimes | Often | Very Often |
| --- | --- | --- | --- | --- | --- |
| 1. You spend a lot of time thinking about social media or planning how to use it | 1 | 2 | 3 | 4 | 5 |
| 1. You feel the urge to use social media more and more | 1 | 2 | 3 | 4 | 5 |
| 1. You use social media to forget about personal problems | 1 | 2 | 3 | 4 | 5 |
| 1. You have tried to cut down on the use of social media without success. | 1 | 2 | 3 | 4 | 5 |
| 1. You become restless or troubled if you are prohibited from using social media | 1 | 2 | 3 | 4 | 5 |
| 1. You use social media so much that it has had a negative impact on your job/studies | 1 | 2 | 3 | 4 | 5 |

**Internet Addiction Improvement Motivation Scale (IAIMS)**

The following questions will assess your motivation levels regarding Internet Addiction. Please read each question carefully and circle a number from 1 to 6, according to the response provided. There are no right or wrong answers. Do not spend too much time on any statement.

|  | Strongly Disagree | Disagree | Somewhat Disagree | Somewhat Agree | Agree | Strongly Agree |
| --- | --- | --- | --- | --- | --- | --- |
| 1. I contemplated effects of internet use on me | 1 | 2 | 3 | 4 | 5 | 6 |
| 1. I recognize the positive effects of appropriate internet use are much larger than those of excess internet use | 1 | 2 | 3 | 4 | 5 | 6 |
| 1. I have many resources to succeed in reducing Internet use | 1 | 2 | 3 | 4 | 5 | 6 |
| 1. I want to progress necessary stages for reducing internet use | 1 | 2 | 3 | 4 | 5 | 6 |
| 1. I let others know my attempt to control internet use | 1 | 2 | 3 | 4 | 5 | 6 |
| 1. I want to receive professional help to reduce internet use. | 1 | 2 | 3 | 4 | 5 | 6 |
| 1. I think continuous internet use does not matter | 1 | 2 | 3 | 4 | 5 | 6 |
| 1. I did not contemplate my internet use problem | 1 | 2 | 3 | 4 | 5 | 6 |
| 1. I feel various discomforts from my internet use problem | 1 | 2 | 3 | 4 | 5 | 6 |
| 1. I do not want to reduce my internet use | 1 | 2 | 3 | 4 | 5 | 6 |

**Adapted Stages of Change Questionnaire**

The following questions describe your social media use. Please read the following statements carefully and write your response on the right column (alphabet from A to F). Please tick one response only. There are no right or wrong answers. Do not spend too much time on any statement.

| Did you use social media for more than 20 hours a week in the past month? | Response |
| --- | --- |
| A: Yes, and I do not plan to reduce my social media use |  |
| B: Yes, and I plan to reduce my social media use in the next 6 months |  |
| C: Yes, and I plan to reduce my social media use in the next 30 days. |  |
| D: Not this month, but I have used social media for less than 20 hours a week in the past 6 months |  |
| E: No, and I have not used social media more than 19 hours a week in the past 6 months |  |
| F: No, and I have NEVER used social media more than 19 hours a week |  |

**Screen time Application/Digital Wellbeing Application**

The following table represents your objective social media usage for a week. Please refer to your Screen time Application for iOS mobile users and Digital Wellbeing Application for Android mobile users respectively. Fill in the total social media usage on your mobile devices (smartphones and tablets) in the table below in minutes.

| Total social media usage  (minutes): |  |
| --- | --- |

**References**

Andreassen, C. S., Billieux, J., Griffiths, M. D., Kuss, D. J., Demetrovics, Z., Mazzoni, E., & Pallesen, S. (2016). The relationship between addictive use of social media and video games and symptoms of psychiatric disorders: A large-scale cross-sectional study. *Psychology of Addictive Behaviors*, *30*(2), 252.

Apple Inc. (2023). *iPhone User Guide : Keep track of your screen time on iPhone*. Retrieved March 16, 2023 from <https://support.apple.com/en-sg/guide/iphone/iph24dcd4fb8/ios>

Faust, K. A. (2017). *Applying the transtheoretical model to problematic digital game use*. University of Rhode Island.

Google LLC. (2023). *Digital Wellbeing*. Retrieved March 16, 2023 from <https://play.google.com/store/apps/details?id=com.google.android.apps.wellbeing&hl=en&gl=US>

Pakpour, A. H., Fazeli, S., Zeidi, I. M., Alimoradi, Z., Georgsson, M., Brostrom, A., & Potenza, M. N. (2022). Effectiveness of a mobile app-based educational intervention to treat internet gaming disorder among Iranian adolescents: study protocol for a randomized controlled trial. *Trials*, *23*(1), 1-13.

Park, J. W., Park, K. H., Lee, I. J., & Kwon, M. (2012). Standardization study of internet addiction improvement motivation scale. *Psychiatry investigation*, *9*(4), 373.

Pontes, H. M., & Griffiths, M. D. (2015). Measuring DSM-5 internet gaming disorder: Development and validation of a short psychometric scale. *Computers in Human behavior*, *45*, 137-143. <https://doi.org/10.1016/j.chb.2014.12.006>
